# Supplementary material for: Molecular Typing of Mastadenoviruses in Simultaneously Collected Nasopharyngeal Swabs and Stool Samples from Children Hospitalized for Acute Bronchiolitis, Acute Gastroenteritis, and Febrile Seizures
Source: Microorganisms. 2023 Mar 17;11(3):780. doi: 10.3390/microorganisms11030780 (PMC10058226; doi:10.3390/microorganisms11030780)
Supplement: Supplementary file 1 [file microorganisms-11-00780-s001.zip › microorganisms-2208029-supplementary.pdf]

Table S1: HAdV genotype frequencies in NP swabs and stool samples obtained from children with acute bronchiolitis (AB), acute gastroenteritis (AGE), febrile seizures (FS), and healthy controls (CO) at the initial examination and at the follow-up examination.

Group	AB		AGE		FS		CO		Follow-up		All		
Genotypes (no.)	NP	Stool	NP	Stool	NP	Stool	NP	Stool	NP	Stool	All	NP	Stool
B3	1	1	0	0	1	1	0	0	0	0	4	2	2
C1	0	1	0	1	6	5	0	0	2	3	18	8	10
C2	0	2	5	4	5	7	1	1	4	10	39	15	24
C5	1	2	2	2	2	1	0	0	1	1	12	6	6
C6	0	0	2	2	1	1	0	0	1	1	8	4	4
F40	0	1	0	2	0	0	0	0	0	0	3	0	3
F41	0	1	0	15	0	3	0	0	0	3	22	0	22
A31	0	0	0	0	0	1	0	0	0	0	1	0	1

HAdV = human mastadenovirus, AB = acute bronchiolitis, AGE = acute gastroenteritis, FS = febrile seizures, CO = control group, NP = nasopharyngeal swab.
